# Women and their birth partners’ experiences following a primary postpartum haemorrhage: a qualitative study

**DOI:** 10.1186/s12884-016-0870-7

**Published:** 2016-04-18

**Authors:** T. Dunning, J. M. Harris, J. Sandall

**Affiliations:** King’s College Hospital Foundation Trust, London, UK; Florence Nightingale Faculty of Nursing and Midwifery, King’s College London, London, UK; Women’s Health Academic Centre, King’s Health Partners, Division of Women’s Health, King’s College London, London, UK

**Keywords:** Postpartum haemorrhage, Birth trauma, Obstetric emergency, Childbirth, Post-traumatic stress disorder, Birth partners

## Abstract

**Background:**

Postpartum haemorrhage (PPH) is a common obstetric complication. Rates of PPH are increasing in a number of developed countries. This is concerning as PPH is recognised as a leading cause of maternal morbidity and mortality which includes psychological and emotional distress. There is limited understanding of the emotional impact of PPH experienced by women and their birth partners. This study qualitatively describes the experiences of women and their birth partners who experienced a primary PPH.

**Methods:**

Semi-structured interview study. Couples were recruited via maximum variation sampling, which, by purposive sampling drew participants from three groups depending on the degree of PPH: minor (500–1000 ml), moderate (1000–2000 ml) and severe (>2000 ml). Interviews took place from 4 to 14 months post birth, and data were analysed via Framework analysis.

**Results:**

In this qualitative study, 11 women and six partners were interviewed. Data were organised into four interrelated themes; Control, Communication, Consequence, Competence. Just over half of the women and their birth partners were unaware they had a PPH, and would have preferred more information either at the time or in the postnatal period. The findings suggest that birth partners also required more information, especially if separated from their partner during the PPH.

**Conclusions:**

This study provides valuable insights into women’s reports of their feelings and experiences during and after a PPH, and how their partners feel having observed a PPH. This study suggests that women who have had a PPH of any volume would like more information. Further investigations into the timings, methods and effectiveness of discussions following a PPH are recommended.

**Electronic supplementary material:**

The online version of this article (doi:10.1186/s12884-016-0870-7) contains supplementary material, which is available to authorized users.

## Background

### Patient experience in maternity care

Within the UK, debates over sub-optimal patient experiences in hospitals have gained momentum since the investigation into failings at the University Hospitals of Morecambe Bay NHS Foundation Trust [1] and Mid Staffordshire NHS Foundation Trust Hospital between 2005 and 2009 [2]. While such system wide problems have not focussed on maternity care they have prompted debate over wider matters of patient experiences.

Within maternity care there is increasing attention to listening to patients to improve their experiences, quality and safety. Women have reported that full explanations of what is happening, the presence of a partner, and good communication between staff promote feelings of safety [3]. In contrast, although some women are able to identify when something is wrong in pregnancy, birth and/or postnatally, their concerns are not always taken seriously or acted upon [4, 5].

Postpartum haemorrhage (PPH) is recognised as a leading cause of maternal morbidity and mortality. Although some studies have explored women’s experiences of PPH [6–10], these have focussed on severe PPH. Only one of these studies considered the partner’s views [6]. Within a UK context, a PPH of any volume triggers an emergency protocol involving a multidisciplinary team enacting several procedures in rapid succession. While more severe losses may result in greater physical consequences, it is not yet known if psychological consequences of these interventions are greater or lesser dependent on volume lost. No studies could be identified that explored the experiences of a PPH for less than a 1500 ml loss. There is currently a gap in the literature exploring the views and experiences of women and their partners where a PPH of a lesser volume occurred. This may have implications for follow up care such as psychological and psychical wellbeing for this group of women. In this context, this qualitative study aimed to investigate the experiences of women who have had a primary PPH of varying volumes (major, moderate and minor), and the experiences of birth partners who witnessed the PPH.

## Methods

### Ethical approval

Full ethical approval was gained by the NRES Committee – East Midlands Nottingham 1 (114/EWM/0126). Local Trust Research and Development approvals were obtained prior to the start of the study.

### Sample

Inclusion criteria were women who had an estimated blood loss (EBL) of over 500 ml within the first 24 h of a vaginal birth. Birth partners who were present at the time of the PPH were also invited to participate.

The exclusion criteria excluded couples under 18 years old, those who lacked the capacity to provide informed consent, those who were undergoing formal complaints procedures within the Trust, individuals known to the researcher, and women who had a caesarean section (as the management of PPH at caesarean section is somewhat different to PPH management following a vaginal birth). Women without birth partners, or those whose birth partners did not wish to take part, were eligible to be interviewed. However, birth partners who wished to participate when the woman declined participation were excluded to protect the anonymity of the woman.

### Recruitment

Women and birth partners were selected through maximum variation sampling at a large London teaching hospital. An a priori aim of recruiting a minimum of two couple dyads of minor, moderate and severe loss was set. Data saturation was not an a priori intention. Recruitment ceased following successful interviews of six partners.

Potential interviewees were identified from hospital records of a PPH from the previous year (employing the definition of PPH as used in the RCOG [11] guidelines), and contacted by a consultant midwife. Those who expressed interest in participation were contacted by the researcher (TD), a midwife based at another Trust who was previously unknown to them, and an interview appointment made. Recruitment occurred between 6 weeks and 18 months post birth. Various studies have demonstrated sound recall of birth events beyond 18-months of birth [12]. It was felt interviewing before 6-weeks would (i) have interrupted the initial parenting experience for new mothers and (ii) would not have allowed enough time for women to process the experience sufficiently. All births occurred in 2013 and 2014.

### Design and procedure

This study adopted an exploratory, semi-structured interview design, which can be seen in Additional files 1 and 2. The interview was successfully piloted with a maternity service user. The interview structure was not amended following the pilot. All interviews were conducted at the participants’ homes and the length of interviews ranged from 10 to 43 minutes. Five of the couples chose to be interviewed together, while one couple chose to be interviewed separately. Following signing of a consent form, the interview continued via the predesigned interview schedule. Interviews were audiotaped, anonymised and transcribed using an approved transcribing service.

### Analysis

The contents of the transcripts were analysed for emergent themes and coded using the matrix-based thematic method developed by the National Centre for Social Research, Framework Analysis [13]. This approach enables a systematic and transparent analysis of the material while facilitating the use of pre-existing empirical evidence in the design and analysis stages. It can be adapted to research with specific questions, and is suitable for research within a limited timeframe [14]. After initial familiarisation with the data set, including listening to recordings and reading the transcripts several times, a coding scheme was developed. This was used to code the data, using NVivo Version 10, which is optimised for the Framework approach. An iterative approach was used, so that the coding scheme was adjusted during analysis. After codes were finally assigned, the data were systematically summarised into matrices to aid identification of recurrent between-participant themes. A thematic analysis was conducted using the summarised data within the matrices [15] with comparisons made both within and between cases.

### Validity

This study took the following steps to minimise bias and increase reliability, in accordance with Yardley [16]. A new mother and recent user of maternity services provided comments on the study protocol and interview guide. Reflexivity – the sensitivity to the way a researcher has shaped the collection and analysis of the data [17] was employed, acknowledging that the responses could have been interpreted from a clinician’s point of view, and the researcher was careful not to jump to conclusions or make assumptions about the participants’ experiences. Furthermore, two coders (TD and JH) independently analysed the transcripts.

## Results

### Participants

Of the 17 women invited to participate, 11 women and six partners agreed to take part in the study. Six women declined to participate; no follow up questions were asked about their reasons for declining. All partners had been present at the birth and subsequent PPH. Of the five women who were interviewed separately, one had no birth partner present, two had birth partners that declined to participate, and two birth partners were unable to participate.

### Background characteristics

Table 1 provides a summary of participants’ demographic information. There was a wide range of ethnicities across the 11 women, the mean age of the women at the time of interview was 32 years and they were from different socio-economic backgrounds.

**Table 1:** Socio-demographic and obstetric details (names anonymised)

EBL (ml)	Women’s details			Partner details		Labour information		
	Female participants	Age range (yrs)	Parity	Male participants	Age range (yrs)	Mode/Place of birth	Other factors	Months since birth
600	W1	30–34	1	M12	40–44	SVB/OLU	MROP	4
600	W2	30–34	1			SVB/MLU	MROP	5
680	W3	20–24	1			Forceps/OLU		13
1100	W4	30–34	1	M13	30–34	SVB/OLU	Twins, preterm 2^nd^ PPH	10
1265	W5	30–34	5			SVB/OLU		6
1570	W6	35–39	1	M14	30–34	Forceps/OLU	2^nd^ PPH	5
2200	W7	30–34	1	M15	40–44	Ventouse/OLU		10
2200	W8	30–34	2			Forceps/OLU	VBAC	4
2200	W9	35–39	1			SVB/OLU	Preterm	8
2300	W10	30–34	1	M16	30–34	SVB/MLU		14
2560	W11	30–34	1	M17	30–34	Forceps/OLU		5.5

*MLU* midwifery led unit, *OLU* obstetric lead unit, *SVB* spontaneous vaginal birth, *MROP* manual removal of placenta, *VBAC* vaginal birth after caesarean, *2nd PPH* secondary PPH

For most women, it was their first child birth (*n* = 9). Six women had spontaneous vaginal births (SVB) and five had instrumental births (1 x ventouse and 4 x forceps deliveries). Two women gave birth in a midwifery led unit, and nine in an obstetric led unit. No women gave birth at home or in water. Some women had other complications such as preterm birth (*n* = 2), manual removal of placenta (*n* = 2) and secondary PPH (*n* = 2). The median length of time of the interview since birth was 6 months, with a range from four to 14 months (see Table 1).

Participants were divided into the minor, moderate or severe group (in line with RCOG [11] definitions) depending on the estimated volume of blood lost.

**Fig. 1:**
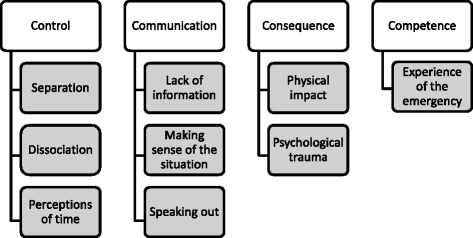
Key themes emerging in interviews

Following analysis four main themes emerged, each with sub-themes (Fig. 1).

#### Control

The theme of control contained three subthemes: separation, dissociation and distorted perceptions of time, discussed below. The majority of women interviewed felt a sense of control during the emergency, ascribing this to the care they received:

*“Everything for me went by so quickly, and then when I was in the hands of their care I felt OK.” – W10 (Severe)*

*“No, I wasn’t scared, I wasn’t scared. When I found out I wasn’t scared, I wasn’t scared, I thought it’s going to happen with me or happen to my baby. But they were really trying. They tried their best, they tried. I think it was about seven or eight doctors round me, and a lot of doctors around her. So I was happy, I was like, it’s what will be will be, you know.” – W9 (severe)*

However, two women – both from the moderate loss group – discussed a sense of losing control:

*‘I felt like I had no control and that they didn’t know how I was feeling and … whether they were going to be able to deal with the issue. Obviously I knew that they could because they were a hospital, but in that second in that situation all sorts runs through your head’ – W5 (Moderate*)

‘*I mean I was so out of it. I was so out of it’* – *W6 (moderate) (speaking about the time of the PPH)*

##### Separation

Separation from each other and from the baby was identified as a difficulty for both women and their birth partners when the woman was moved from the delivery room to another area of the hospital or to the operating theatre. One partner talked about being sent home from the labour ward half an hour after his wife had come out of theatre, and how he had found this very distressing. Two birth partners talked of being left with their new-born with no support or no one telling them what was going on:

*‘…they rushed her off and just left me with the baby, um, the baby, and I was asking what was wrong and all they could say was, ‘She’s lost a lot of blood, we don’t know why, we can’t stop the bleeding.’ Um … and … yeah, so I was left with the baby and in … yeah, just in a daze really.’- M16 (Severe)*

Separation was also an explicit concern for three of the 11 women, but in relation to being separated from their new born. One woman who was taken to the operating theatre spoke of her sadness at being separated from her baby:

*‘I’m really sad that I missed that part of seeing her being weighed and all that’ – W1 (Minor- who was taken to theatre for manual removal of placenta)*

##### Dissociation

Ozer et al. [18] define peri traumatic dissociation as ‘unusual experiences during and immediately after the traumatic event, such as the sense that things are not real’ (p. 1700). Dissociation was a consistent finding across the three groups interviewed, suggesting that it may not be the severity of the PPH that is so important in long-term recollection; but other factors help people feel in control or otherwise:

*“I couldn’t tell anything, I was knocked out completely” – W3 (Minor)*

*“I mean I was so out of it. I was so out of it.” W6 (Moderate)*

*‘I was so out of it that … I didn’t really think, I didn’t even know what was going on really’ – W7 (Severe)*

##### Distorted perception of time

Another consistent finding across the three groups indicates a feeling of distortion of time. Two women went to the operating theatre for further exploration, which could explain why they may have felt confused or anxious, however one woman was in the room with her baby and partner at the time:

*‘Yeah, do you know what, the time went really, really quickly, I thought I was in there for 20 min, but when I came out Andy told me I was in there for an hour-and-a-half.’ – W10 (Severe) - (In the operating theatre)*

*‘I was really unprepared for that so my legs were still in stirrups, because I kind of thought after she came out I could just relax and cuddle her and lie there and sort of feel like it was all in the past, and I wasn’t expecting this … it felt like an hour or more. I don’t know how long it was, and I don’t know what’s normal. But that just, that, that part went on and on and on’ –W6 (Moderate) –(In room)*

#### Communication

Communication emerged as an important theme. More than half of women and their partners were unaware of the PPH at the time and gave examples of where information about what was happening or its implications was lacking either during or after the emergency.

##### Lack of information

A consistent finding across the three groups was of the participating women being unaware they had lost more blood than average both at the time and after birth. Some women did not know they had lost more blood than average until contacted to take part in this research. One partner who was present throughout the PPH thought it was a normal part of the birth:

*‘I didn’t even know, I thought most women got, um, iron tablets so it wasn’t a, it wasn’t a big deal at that point. Um, I thought that was just the norm, all women lose blood and …’ M16 (Severe)*

*‘Um … and … but as far as … I wasn’t really aware that I was, I did haemorrhage, to be honest, the first I really knew about it was when they gave me, um, iron tablets.’ – W4 (Moderate)*

*‘I think I was a bit surprised I hadn’t been told, but to be honest it wouldn’t have changed how I felt or how I recovered or … it wouldn’t have had any impact, it would have just been another bit of information’– W11 (Severe)*

Women expressed a wish for more information in the postnatal period, one woman spoke of her difficulties in breast feeding (resulting in her baby being readmitted to hospital) and only found out weeks later this could have been because of her severe blood loss:

*‘it was only having spoken to midwives and sort of health visitors afterwards they were a bit like, ‘Well yes, because you had that blood loss that’s, that can happen.’ And I think if we’d been aware of that, um, that would have been really, really useful’ –W11 (Severe)*

However, one woman acknowledged that information might have caused more distress and fear if given at the time. This proves an interesting insight into information giving practices, especially around timing:

*‘I guess the, the idea is that they don’t want to scare you immediately so they don’t, they’re like quite soft touch with the information, but … I’m not, I would prefer … all of the information, but I guess it depends on the person, so I guess they go with like a … kind of … ‘Oh you’ve lost, you know, a little bit more blood than usual,’ W7 (Severe)*

Two birth partners said they wanted more information at the time of the PPH; however, this was only the case for men whose partners were taken to the operating theatre. Men who were present in the room at the time of the PPH said they would have liked more information, but acknowledged that this might have added to confusion and distress, echoing what the women reported:

*‘I would have liked more information about what … there were lots of things running through your head but the main thing immediately is, is she OK? What caused that? … What does that mean now for her immediate, immediate situation? Is she all right, what do we need to do to make it better? I didn’t really get a lot of information on that’ –M16 (Severe) - Partner taken to theatre*

*‘I mean I would have, because they have to make a judgement, a judgement, don’t they, as to what is too much information and when, what is just the essential information. So for me I think we could have, er … done with a little bit more, but it’s difficult because everyone’s in such a, we, the couple are in such a heightened emotional state and the more traumatic it gets the more emotional it becomes, so you can’t really give them too much information because it will just confuse you’ M14 (Moderate) - PPH in the room*

##### Making sense of the situation

Women who had experienced a severe PPH had tried to make sense of the emergency once back home. Some adopted information seeking behaviours afterwards, for example, using the internet as a means of making sense of their situation:

*‘So it wasn’t until I was at home, it may have been a week or two afterwards that I Googled what it was, that I realised, and I hope my understanding is correct, I think at the time I thought it was something to do with the tear and the stitches, and then I realised it was actually more to do with the placenta, and it … So I was … until I Googled it myself I was completely unaware of what had actually happened.’ – W11 (Severe)*

##### Speaking up

One partner was very adamant about ‘speaking up’ for his wife. He was concerned midwives and doctors could be missing warning signs of deterioration following the blood loss so he had told each midwife who was caring for his wife the story:

*‘But I definitely wanted to speak to everybody there to make sure they were all aware of what had happened so that, um … (W10 severe) got the best level of care during the night and there wasn’t anything missed.’ –M16*

One woman asked her partner to go and get a doctor, even though the midwife was present, when she started to feel unwell:

*‘Oh you’re just … tired. You just need a rest.’ (they said). And I just didn’t think that’s what it was. So I asked (M15) to insist on the doctors coming back in. And then they did come back in’ – W7 (Severe)*

#### Consequence

This theme addressed the physical and psychological implications for having a PPH, broken down into two subthemes.

##### Physical impact

It took most women a long time to recover physically, regardless of the volume of blood they had lost. Women spoke of being on iron tablets for months after birth, and found everyday activities, such as walking. difficult. These women were being followed up by their GP:

*‘And I was trying to kind of go for walks and stuff but it was just exhausting for me to go for a stroll or something’ W11 (Severe)*

*‘but when I got home I really noticed the strain that the birth and everything had taken on my body and … also my parents came to visit to see the baby and they said that I looked very pallid’ – W2 (Minor)*

##### Psychological impact

Most women had made a good psychological and emotional recovery from their birth experiences. However, women who were in the moderate group reported more birth trauma than those in severe or minor group.

One woman (in the moderate group) reported feeling traumatised following her PPH. Another woman spoke of a breakdown in her relationship with her partner, and attributed part of this to the birth, indicating the possible long-term effects of a traumatic birth:

*‘It’s once I start thinking about it, it like … kind of brings the whole thing back.’ –W6 (Moderate)*

*‘No, I’m not with … I think maybe it was too much for him.’ W5 (Moderate) - speaking about her relationship with her partner following the birth*

Interestingly women in the severe group appeared to cope well emotionally following their PPH, even though they had lost the most blood and had generally been admitted to a High Dependency Unit:

*‘I thank God I gave birth at [the hospital]. Yeah. That was a good thing. And the horrible part of it is … if you are kind of, anyway, I can say that it was good, if you are kind of sick and somebody rescue you it’s not bad, it’s still good. I can’t say it’s bad because I have myself happy, I have my baby happy, we’re happy, we’re going home happy’– W9 (severe)*

One partner experienced emotional and psychological distress in the months following the birth. He had sought help from his GP and counselling services:

*‘definitely I felt numb, just …yeah, emotionally I think it did affect me more than her, um, because … all, yeah, and it did so for a number of, definitely, six weeks and even beyond … I felt a different person, it was like I had changed and I just felt like … um … yeah because it just was there in front of you for such a long period and it kind of, the shock and the … and me and (W10) were talking and she’d say, ‘Maybe you should go and get some counselling or … that would be helpful’ – M16 (Severe).*

#### Competence

This theme emerged throughout the interviews. Mostly women were pleased with how the PPH was managed at the time, and the actions of the midwives and obstetricians were perceived as competent.

##### Experiences of the management of the emergency

Women with severe PPHs were generally pleased with the management of the emergency. They spoke of people coming in the room, but generally feeling well looked after and safe. Staff appeared competent and confident, as one mother recalled:

*‘No, the care up to that point had been so good and everybody had been, um, so supportive, not that I’d expected otherwise but I was quite, um, pleased with the time they took just to reassure me and … that sort of personal touch, do you know what I mean, was really nice’– W11 (Severe)*

However, some treatment, such as requiring a blood transfusion, could be painful and emotionally distressing:

*‘hm, I was, um, scared when they say I had to, they had to do a blood transfusion, because I always say no, I don’t want to have another person’s blood in my body. And when they do that the transfusion was very hard for me. Um, it was very painful for my arm when the, when the midwife come to do the, the thing’. – W8 (Severe)*

## Discussion

These findings provide insight into women’s and their birth partners’ experiences of PPH. One key finding was that some women across the three groups were unaware they had had a PPH, and many expressed a desire for more information in the postnatal period. Partners too wished for more information.

## Control

Overall, the level of control felt by women was variable, two of three women who experienced a minor PPH reported feeling out of control, while two of the five women who had a severe PPH reported feeling in control, supporting Beck’s [19] view that ‘birth trauma is in the eye of the beholder’ (p.28). This present study also supports the findings of Thompson et al. [7], as women with severe PPH felt more satisfied with their care and expressed feelings of being more in control compared to those in the moderate and minor groups. This could be explained by their perception that the emergency was responded to appropriately as evident by the number of doctors and midwives attending the emergency. These women may have received more intensive follow up care and information following a severe PPH. This did not seem to be the case in this study, although case records were not accessed. Further research investigating the experiences and feelings of women who had a moderate PPH might produce findings that would usefully inform practice such as the content and timing of specific information for them.

Ayers’ [20] study of women’s thoughts and emotions during a traumatic childbirth found that women who experienced dissociation during birth were more likely to develop PTSD symptoms. She also observed that women reported distorted perceptions of time, in both the PTSD and control group (no PTSD symptoms). Dissociation and distorted perceptions of time were a common theme across the minor, moderate and severe groups, even for women who did not report losing control. One hypothesis may be that this was related to medication given at the time of the PPH, or could perhaps be explained by confusion and lack of understanding of the situation; all these possible reasons have implications for communication and information provision.

## Communication

Across the three groups there was evidence that some participants were unaware they had a PPH. This has clear implications for professional practice. Such information may be helpful - for example, knowledge about the risk of severe blood loss impacting on breastfeeding - or implications for future birth planning. Psychological debriefing is a treatment used to reduce psychological distress following a traumatic event [21]. The evidence related to postnatal debriefing has been much explored [21, 22] with mixed reviews of efficacy. Priest et al. [23] found such services were generally ineffective in preventing psychological disorders (PTSD and PND), but Gamble et al. [24] concluded that debriefing was potentially useful in reducing PTSD symptoms at three months. However, these data do not exclude the giving and explaining of information related to a birth event. The data presented in this present study highlight women’s expressed wish for some or more information about their PPH in the days and weeks after their birth. Similar findings have been found outside maternity settings; for example, a study of patients cared for in an Emergency Department found that many did not understand what care they had received or their discharge information [25]. Further research is required into the optimal timing and effective delivery method of information to women who have experienced a PPH.

## Consequence

The physical effects of having a PPH were highlighted in this study. Women across the three groups described the time it took them to recover physically, for which they were unprepared. This may be related to the lack of information given. Thompson et al. [26] found ‘amongst women intending to breastfeed, those with a higher estimated blood loss were less likely to fully breastfeed in the first week postnatally than women with a lower estimated blood loss’. They suggested women need appropriate and timely support and advice around breastfeeding following a PPH. The data presented here provide some support for this finding, with one woman feeling the PPH had an effect on her breastfeeding. Service providers need to be aware of the potential impact of this emergency on breastfeeding, and should consider how this could be addressed.

## Competence

Across the groups women and their partners felt that the PPH was managed well, and many expressed their appreciation of the skills of the doctors and midwives involved during the emergency. However, as noted in the limitations section below, this study took place in a major teaching hospital and further research could helpfully address the feelings of competence and confidence imparted by health care practitioners to women experiencing a PPH in settings where there is less familiarity with the emergency and its management.

## Partner experiences

This study also provides insights into partners’ experiences of witnessing a PPH and its management. Although the sample of partners was smaller than that of women, a partner was interviewed from each of the minor, moderate and severe groups. All partners interviewed were spouses, and there were no same sex couples. This study’s findings have parallels with Hinton et al’s [27] interview based study in which one partner recalled ‘being left in blood stained delivery rooms for hours’, whilst a partner interviewed in this study recalled being left in the delivery room for considerable time until he was 'found by the cleaners'. Lack of communication is a theme that is interwoven through the literature about partners being present at traumatic births [6, 27, 28]. Lindberg and Engström [28] found that such new fathers felt abandoned, excluded and separated from their partners.

Separation was also a central theme in partners’ experience in this study. Partners physically separated from their wives during the PPH appeared to want more information at the time rather than afterwards. This has practice implications for the ways healthcare professionals give information, and how they address the new father who, is often left ‘holding the baby’. Further research into partners’ psychological health and resilience following their witnessing of a traumatic birth is needed, and strategies for support and identifying PTSD in partners could be developed to ensure this group is not neglected. Currently, around 98 % of male partners attend the birth of their baby in the UK [29]. There is therefore a high likelihood that many men witness a ‘traumatic’ event. Furthermore, it is important to acknowledge that only husbands came forward to be interviewed. There are therefore groups of people who witness traumatic birth events whose voices have not been heard, for example, same sex couples, other relatives, or partners who are no longer living with the mother of their child.

### Strengths and limitations

Recruitment took place from just one hospital. This resulted in comparable experiences, as all participants will have likely received treatment following Trust and national guidelines. We did not explore the influence of patient-level and provider-level effects on patients’ experiences post PPH. The study site was a large referral unit with specialist resources; no woman had to be transferred to another hospital. Research in a smaller, or rural, hospital or women who gave birth at home might have found different patterns of women’s and partners’ experiences.

A further strength of this study was that women with varied blood loss were included and compared. The literature review found no studies where women who had had a minor PPH (500–10,000 ml) had been interviewed and few studies have included participants who had a moderate blood loss (1001–2000 ml) [7]. As this study highlights, this ‘moderate group’ has experiences to share, and their reflections provide some interesting implications for practice.

The sample included women with a range of socio-demographic characteristics with a range of ethnicity, occupational background, and social support. However, all participants were able to understand and speak English, so findings cannot be generalisable to the experiences of all women giving birth in the UK. It is important to note that some participants had other pregnancy/labour complications which may have added to their experience and recollection of the event, such as preterm birth, multiple birth and secondary PPH. It is also recognised that the length of time since birth means that there may have been an element of recall bias.

The voice of birth partners is often unheard in maternity research, and efforts were made to encourage participation here. Four partners were unable or unwilling to participate in the research. One couple requested to be interviewed separately, while the rest requested to be interviewed together. We recognise that the mixture of solo and joint interviews may have impacted on the nature of the data generated in the interviews. The variability in the time period between PPH and interview may have influences the recall of the event.

This study used qualitative methodology, and quality of life/psychological wellbeing were not assessed using validated surveys or questionnaires. More quantitative or mixed method studies would be useful in assessing both short and long term outcomes of experiencing a PPH.

## Implications for practice

Currently, follow up appointments are available for women who sustain perineal trauma (3rd and 4th degree tears) in some Trusts in the UK [30]. The RCOG suggests that ‘It is helpful to review women in the postnatal period to discuss injury sustained during childbirth, assess for symptoms and offer advice on how to seek help if symptoms develop, offer treatment and/or referral if indicated and advice on future mode of delivery’ [30]. This could be applicable to other groups of women who have complicated births, including following a PPH. It may help in the care planning for subsequent pregnancies, as women with history of having a PPH have a three-fold increase of having a PPH in their second pregnancy, compared to women that did not have a PPH [31]. This could address both physical morbidities (e.g. symptomatic anaemia) and psychological morbidities (e.g. symptoms of PTSD) with appropriate referrals. Partners may also be able to be signposted to support services. Whilst this may be resource and time consuming for maternity services, it may reduce anxieties for families, and help improve longer term outcomes, and public health. Such an intervention would need to be defined, piloted, and evaluated, but could have the potential to be implemented for all women as part of the commissioned maternity package or pathway of care. Alternative options could include the development and evaluation of an information leaflet following a PPH. This could outline potential physical and psychological symptoms, an explanation of what happened, and information about support services. This would need to be aimed at both women and partners.

## Conclusion

This study provides valuable insights into women’s reports of their feelings and experiences during and after a PPH, and how their partners feel having observed a PPH. Women and their birth partners require information following an emergency to facilitate planning for subsequent pregnancies and for reassurance. Further research is needed to understand psychological and emotional impacts of PPH of all volumes to determine whether better information and follow up can improve outcomes.

## Additional files

Additional file 1: Interview schedule for women. (DOCX 16 kb) Additional file 2: Interview schedule for birth partners. (DOCX 16 kb)

## Abbreviations

PPH: postpartum haemorrhage
EBL: estimated blood loss
PTSD: post-traumatic stress disorder
PND: postnatal depression
