# Supplementary material for: Women and their birth partners’ experiences following a primary postpartum haemorrhage: a qualitative study
Source: BMC Pregnancy Childbirth. 2016 Apr 18;16:80. doi: 10.1186/s12884-016-0870-7 (PMC4835830; doi:10.1186/s12884-016-0870-7)
Supplement: Additional file 1: — Interview schedule for women. (DOCX 16 kb) [file 12884_2016_870_MOESM1_ESM.docx]

Interview schedule for women

**Part 1 – Welcome**

Introduction, name, position.

Thank you for taking part in this research

Aims and objective of the research.

Reminder that the interview is audiotaped, and will be transcribed.

Reminder about confidentiality, anonymity and withdrawal

Any questions so far?

**Part 2: Birth story**

*(****AIM****: To gain a background into the birth story, capture the recollections of their birth, what was important to them, and what stands out the most*)

Can you tell me about your recent birth?

How was the experience for you?

Was there anything that particularly stood out?

**Part 3 – PPH**

(***AIM****: To understand the experiences of PPH, how it made them feel at the time, what connotations it brings*)

Were you aware of any complications during your birth?

Were you aware of midwives and/ or doctors concerned about blood loss after your birth?

How/ why?

Can you remember how this made you feel?

Did you feel safe?

Did anybody explain to you what was happening?

What was your birth partner doing at this time?

**Part 4 – Looking forward**

*(****AIM:*** *To understand if this had any positive or negative impact on their birth or recollection, suggestions for improvement)*

Have you had the chance to talk to anybody in the maternity services about your experience?

When? Who?

If you were to have another baby, what would you like to be different?

What advice would you give to mothers preparing for childbirth now?

**Part 5– Close**

Is there anything else you would like to add?

Do you have any questions about the research?

Thank you for taking the time to participate in this research.

**Part 6-** **Demographic details**

(***AIM****: To understand the demography of the women being interviewed, and gain useful information about their childbirth that may not be captured in other parts if this schedule)*

Age

What date and time they gave birth

What gestation they gave birth

Where did they give birth? Home/MLU/hospital

Was this their first baby?

If not, how many other children do they have and how old are they?

Was it instrumental delivery/ spontaneous vaginal birth (SVB)?
