# Supplementary material for: Women and their birth partners’ experiences following a primary postpartum haemorrhage: a qualitative study
Source: BMC Pregnancy Childbirth. 2016 Apr 18;16:80. doi: 10.1186/s12884-016-0870-7 (PMC4835830; doi:10.1186/s12884-016-0870-7)
Supplement: Additional file 2: — Interview schedule for birth partners. (DOCX 16 kb) [file 12884_2016_870_MOESM2_ESM.docx]

**Interview schedule for birth partners**

**Part 1 – Welcome**

Introduction, name, position.

Thank you for taking part in this research

Aims and objective of the research - leaflet

Reminder that the interview is audiotaped, and will be transcribed.

Reminder about confidentiality, anonymity and withdrawal

Any questions so far?

**Part 3: Birth story**

*(****AIM****: To gain a background into the birth story, capture the recollections of their birth, what was important to them, and what stands out the most*)

Can you tell me about your recent experience of being a birth partner?

Was there anything that particularly stands out?

**Part 4 – PPH**

(***AIM****: To understand the experiences of PPH, how it made them feel at the time, what connotations it brings*)

Were you aware of any complications during or after the birth?

Were you aware of midwives and/ or doctors concerned about the blood loss after your partner/ relative gave birth?

How/ why?

Can you remember how this made you feel?

What happened next?

Did anybody explain to you what was happening?

Do you feel anything could have been done differently?

**Part 5 – Looking forward**

*(****AIM:*** *To understand if this had any positive or negative impact on their birth or recollection, suggestions for improvement)*

Have you had the chance to talk to anybody about your experience?

If you were to be a birth partner again, what would you like to be different?

What advice would you give to people who are preparing to be a birth partner now?

**Part 6 – Close**

Is there anything else you would like to add?

Do you have any questions about the research?

Thank you for taking the time to participate in this research.

**Part 2-** **Demographic details**

(***AIM****: To understand the demography of the women being interviewed, and gain useful information about their childbirth that may not be captured in other parts if this schedule*)

Age

Gender

Occupation

Relationship to person who gave birth

Have they been a birth partner before?
